# Supplementary material for: High-throughput screening assay for PARP-HPF1 interaction inhibitors to affect DNA damage repair
Source: Sci Rep. 2024 Feb 16;14:3875. doi: 10.1038/s41598-024-54123-8 (PMC10873324; doi:10.1038/s41598-024-54123-8)
Supplement: Supplementary file 1 — Supplementary Information. [file 41598_2024_54123_MOESM1_ESM.pdf]

Supplementary data

High-throughput screening assay for PARP-HPF1 interaction inhibitors to affect DNA damage repair

Saurabh S. Dhakar, Albert Galera-Prat, & Lari Lehtiö*

Faculty of Biochemistry and Molecular Medicine & Biocenter Oulu, University of Oulu, Finland

CONTENT

Table S1 & S2. Statistics of assay validation.

Table S3. Results of the compound screening.

Figure S1. Structural alignment of PARP1 catalytic domain with PARP2-HPF1 complex.

Figure S2. Thermal melting of PARP2 and HPF1 with inhibitors using nanoDSF.

Figure S3. Titration of YFP with CFP-PARP1 and CFP-PARP2.

Figure S4. Dynamic light scattering (DLS) scattering of PARP1/2-HPF1 complexes.

Table S1. Statistics of assay validation at 10 µl volume.

Day and plate number	Minimum Signal (control)			Maximum Signal (FRET pair)			S/N	Z'
	Average	SD	% CV	Average	SD	% CV		
Day 1, plate 1	0.53	0.002	0.33	0.69	0.004	0.59	96.07	0.89
Day 1, plate 2	0.54	0.004	0.71	0.71	0.004	0.59	44.51	0.86
Day 2, plate 1	0.56	0.003	0.50	0.73	0.005	0.70	66.49	0.87
Day 2, plate 2	0.55	0.002	0.43	0.73	0.003	0.37	77.26	0.92
Day 3, plate 1	0.54	0.004	0.72	0.72	0.005	0.73	46.87	0.85
Day 3, plate 2	0.54	0.003	0.64	0.71	0.003	0.46	52.78	0.89

Table S2. Statistics of assay validation at 20 µl volume.

Day and plate number	Minimum Signal (control)			Maximum Signal (FRET pair)			S/N	Z'
	Average	SD	% CV	Average	SD	% CV		
Day 1, plate 1	0.56	0.004	0.63	0.71	0.004	0.59	43.20	0.85
Day 2, plate 1	0.55	0.004	0.69	0.71	0.005	0.70	42.12	0.84
Day 2, plate 2	0.55	0.004	0.65	0.70	0.003	0.37	44.02	0.88
Day 2, plate 3	0.54	0.004	0.78	0.70	0.004	0.53	36.61	0.85
Day 3, plate 1	0.54	0.005	0.97	0.71	0.009	1.25	32.36	0.75

Table S3. Results of the compound screening.

Number of compounds	1832
Filtered compounds (20% fluorescence filter)	1462
Mean of % activity	100.2
Std. Deviation	6.08
Hit limit (% activity)	70.25
Hit compounds	3
Selected after binding study (nano DSF)	2
Inhibition of PARP1/2 activity	2

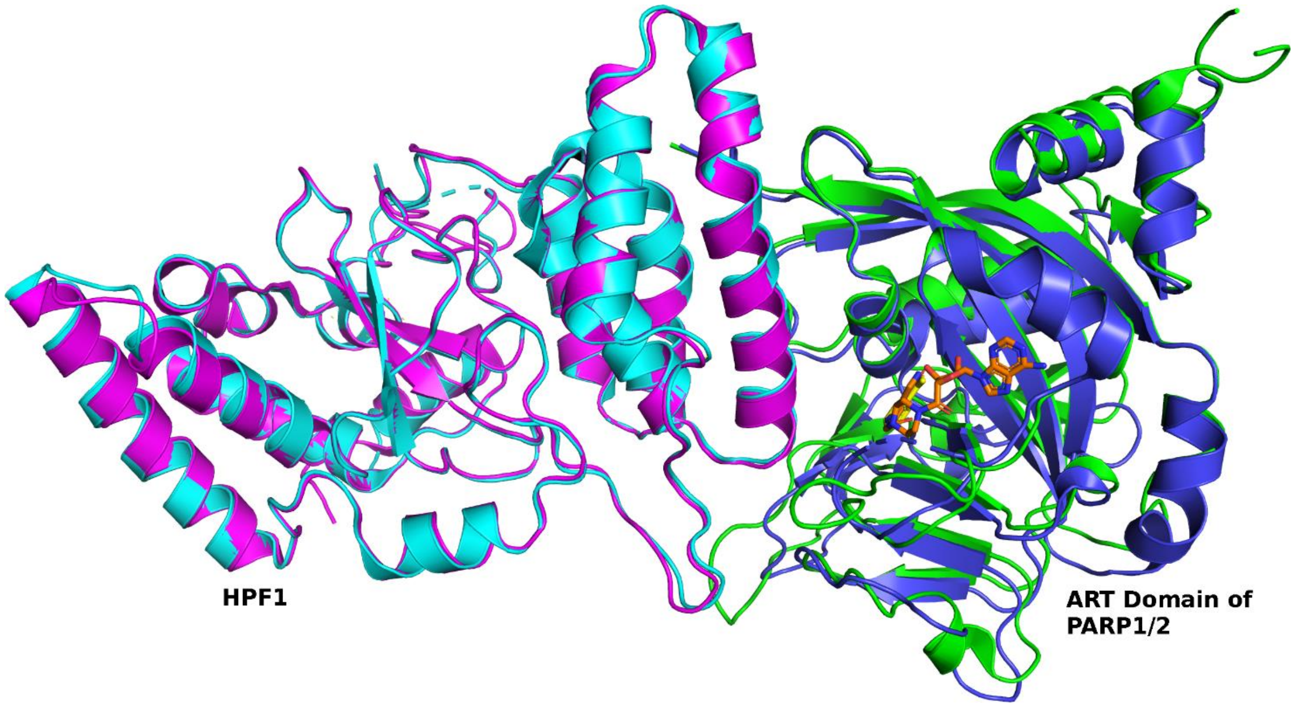

Figure S1. Structural alignment of PARP1 (ART)-HPF1 (PDB id: 6M3I) with PARP2 (ART)-HPF1 complex (PDB id: 6TX3). The ART domain of PARP1 (blue) and PARP2 (green) are interacting with HPF1 (magenta / cyan) with same interface.

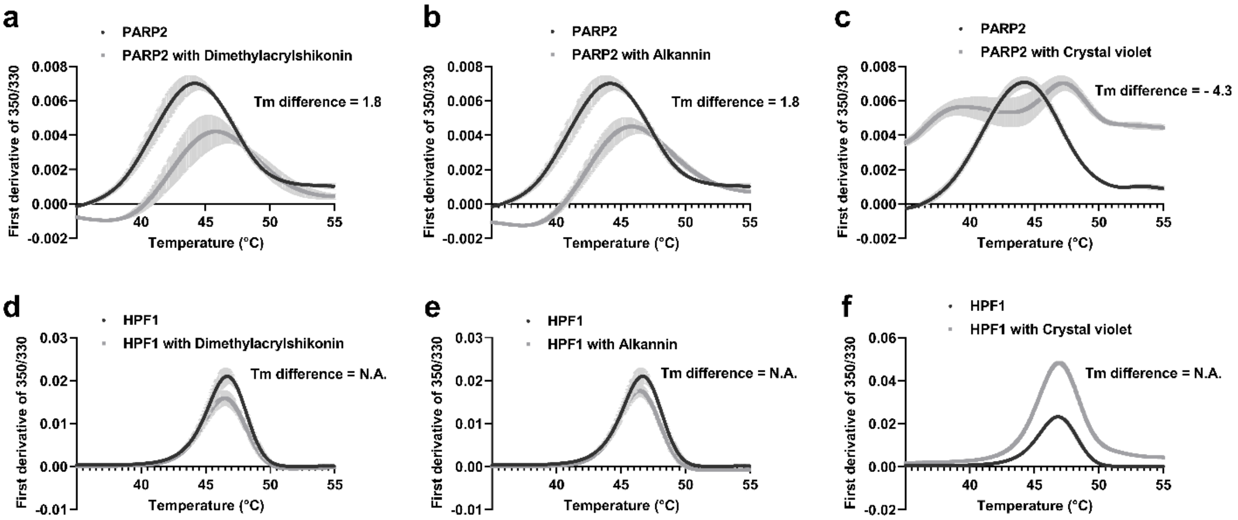

Figure S2. Thermal melting of protein (1 mg/ml) using nanoDSF showing the effect of hit compounds (100 µM) on thermal stability of (**a-c**) PARP2, (**d-f**) HPF1. The data shown are mean ± standard deviation from three independent measurements each with 3 internal replicates. [N.A.: not a significant shift in Tm]

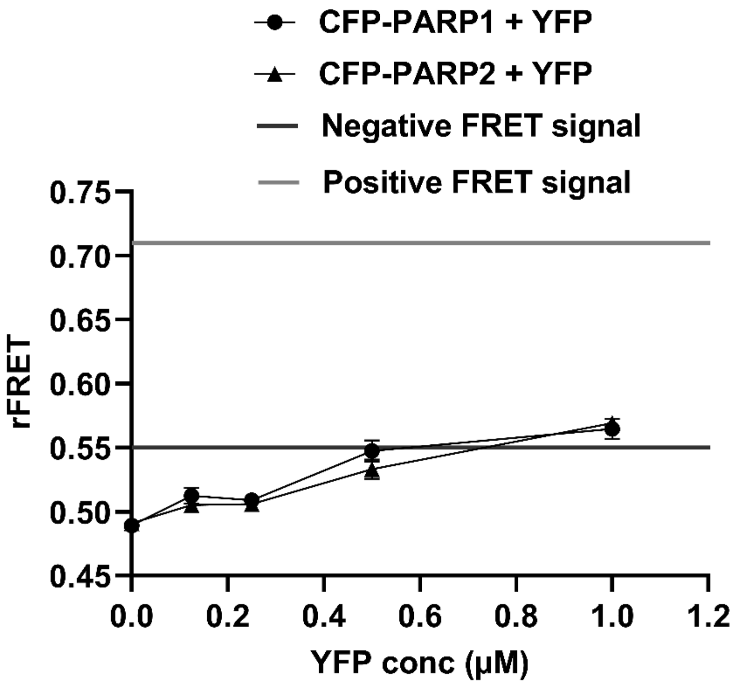

Figure S3. Titration of CFP-PARP1 (0.4 µM) and CFP-PARP2 (0.4 µM) with YFP (0-1 µM) to test the unspecific interaction. In the optimized buffer conditions, the 400nM CFP-PARP1/2 were titrated with YFP (0 – 1 µM) protein to measure the unspecific rFRET signal. The maximum rFRET signal value during the YFP titration was 0.57, which is close to the control rFRET signal (0.55) [FRET control values 0.55 and 0.71 are obtained from supplementary table 2]. This indicates that higher rFRET signal is a result of PARP-HPF1 interaction and not coming from unspecific interaction. All the titration reactions were performed in 20 µl volume in 384-well plates and the data shown are mean with standard deviations of 4 replicates.

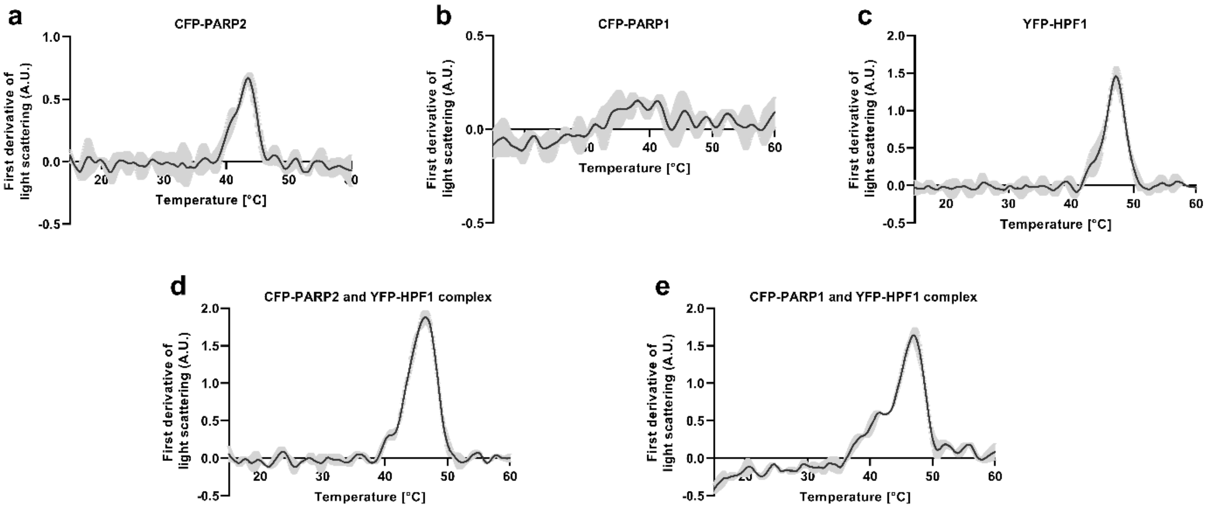

Figure S4. Dynamic light scattering (DLS) scattering using nanoDSF showing the effect of temperature on aggregation of (a) CFP-PARP2 [0.5 mg/ml], (b) CFP-PARP1[0.5 mg/ml], (c) YFP-HPF1[1.0 mg/ml], (d) CFP-PARP2 and YFP-HPF1 complex and (e) CFP-PARP1 and YFP-HPF1 complex. The data shown are mean ± standard deviation from three independent measurements.

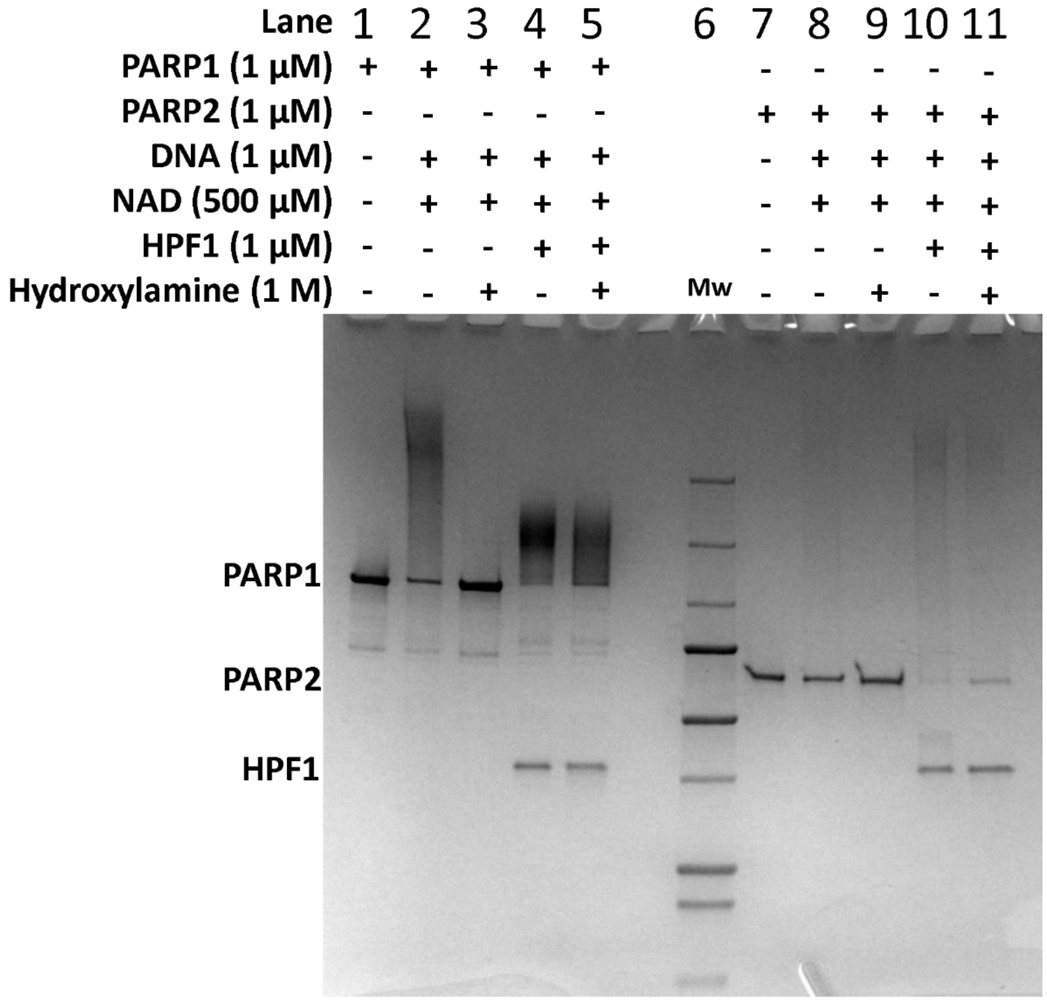

Figure S5. PARP activity assay on SDS-PAGE. PARP1 or PARP2 (1 µM) was incubated with central nicked double stranded dumbbell DNA (1 µM) for 10 min at room temperature (RT). As indicated HPF1 (1 µM) followed by 500 µM NAD^+ was added for 5 min and reactions were stopped using PARP inhibitor Olaparib (500 µM). Reactions were treated with 1 M hydroxylamine (NH_2OH) for one hour as indicated. Reactions were resolved on 4 - 20% SDS-PAGE and stained using blue stain.
